# Evidence for a 12 kDa “Carrier Protein” for Natriuretic Hormone

**DOI:** 10.3389/fendo.2014.00196

**Published:** 2014-11-19

**Authors:** Harvey C. Gonick

**Affiliations:** ^1^Division of Nephrology, Department of Medicine, David Geffen School of Medicine, University of California Los Angeles, Los Angeles, CA, USA

**Keywords:** natriuretic hormone, carrier proteins, hormones, hypertension, review, Anf, proANF

## Abstract

The search for the elusive Na-K-ATPase-inhibiting natriuretic hormone continues. In this review, evidence is presented that isolating the carrier protein for natriuretic hormone from hypertensive plasma is a necessary first step before splitting off the final hormone. The carrier protein has a molecular weight of 12 kDa while the final hormone has a molecular weight of 408 Da. Both compounds inhibit Na-K-ATPase but the compound containing the carrier protein predominates. The question has been raised as to whether the carrier protein is in actuality proANF, a 17 kDa protein that can be split between a 14 kDa protein (the presumptive proANF) and the 3 kDa ANF.

## Introduction

Circulating inhibitors of sodium-potassium adenosine triphosphatase (Na-K-ATPase) have been shown to be of possible pathogenetic importance in the mechanism of essential hypertension (1–3). Although previous studies have demonstrated the presence of both high-molecular weight (HMW), ranging from 11 to 70 kDa (4–8) and low-molecular weight (LMW) either natriuretic or Na-K-ATPase inhibitors, no previous attempts had been made to ascertain whether HMW or LMW forms predominate in hypertension. This review summarizes the steps taken by our laboratory to first identify the HMW form, and then split off the final LMW form of the hormone. We have in the process determined the approximate molecular weight of the HMW form and the precise molecular weight of the LMW form. Unfortunately, while awaiting the identification of the latter compound, it was lost due to freezer failures in two different laboratories a continent apart. This review is presented in intricate detail in the hopes of encouraging subsequent investigators to pursue the final identification of the LMW natriuretic hormone, as well as the identity of the “carrier protein.”

## Predominance of HMW Plasma Na-K-ATPase Inhibitor in Hypertension

**Figure 1:**
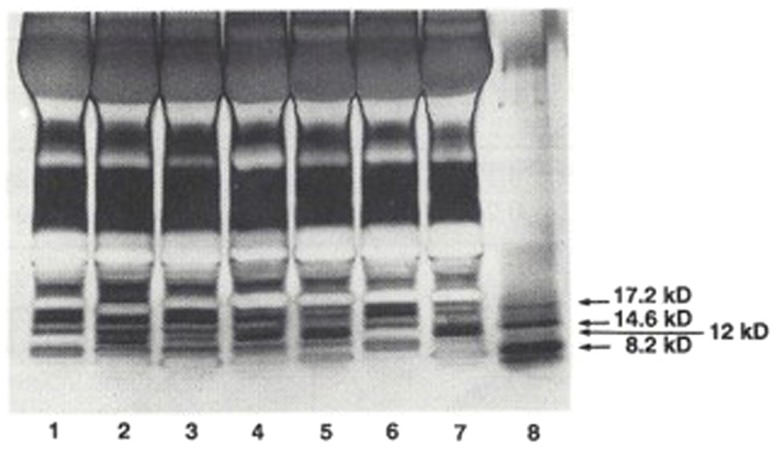
**SDS-PAGE separation of protein bands from plasma from hypertensives and normotensives**. Bands were stained with silver nitrate. Myoglobin protein markers of 17.2, 14.6, and 8.2 kDa molecular weight and the locus of the 12 kDa protein are shown in Lane 8. Lane 1: Normotensive control. Lanes 2 and 3: normotensives with family history of hypertension. Lanes 4 and 5: hypertensives. Lane 6: normotensive control. Lane 7: hyperaldosteronism. Plasma samples applied in lanes 2 and 3 were obtained from the two individuals shown to possess elevated levels of HMW Na-K-ATPase inhibitor. From Ref. (9) with permission from the editors.

In an initial study (9), plasma samples obtained from 26 patients with essential hypertension, 12 normotensive controls, and 6 normotensives with a family history of hypertension were separated into HMW and LMW moieties by passage through a 1 kDa Amicon membrane. The LMW moiety was separated on C-18 Sep-Pak cartridges, applying a 10% stepwise acetonitrile trifluoroacetic acid gradient. The HMW moiety was further separated on Sephadex G-75. Sodium dodecyl sulfate polyacrylamide gel (SDS-PAGE) electrophoresis revealed that the fraction with inhibitory activity contained a distinct 12 kDa protein band, with staining intensity depending on the presence or absence of hypertension (Figure 1). Na-K-ATPase inhibitory activity was found in several LMW fractions, but differences between hypertensives and normotensives were observed in only the 50% acetonitrile fraction (0.29 ± 0.12 SD versus 0.11 ± 0.12 μmol/L ouabain equivalents, *p* < 0.01). Na-K-ATPase inhibitory activity in the HMW fraction was 38 times the inhibitory activity in the LMW fraction and was significantly increased in hypertensives as compared to normotensive controls (10.9 ± 8.9 versus 1.3 ± 0.8 μmol/L ouabain equivalents, *p* < 0.01). Inhibitory activity in both HMW and LMW factions correlated positively with mean blood pressure (*r* = 0.42, *p* < 0.05 and *r* = 0.35, *p* < 0.05). The inhibitory activity in the HMW fraction, but not the LMW fraction, also correlated positively with diastolic blood pressure and inversely with the natural log of plasma renin activity (*r* = 0.40, *p* < 0.01). These results indicate that the HMW moiety is the predominant circulating form of the Na-K-ATPase inhibitor in hypertension.

## Dissociation of the LMW Na-K-ATPase Inhibitor from the HMW Protein Inhibitor

Pooled blood samples from 10 patients with well-documented essential hypertension, not taking any medications for at least 3 weeks, were collected into chilled vacutainers containing sodium ethylenediamine tetraacetic acid (EDTA) and Trasylol (10). Individual samples were also collected from patients with primary aldosteronism, congestive heart failure (CHF), before and after treatment, and normal controls. The treatment of congestive failure employed diuretics and vasorelaxants but avoided digitalis glycosides.

SDS-PAGE was performed according to the procedure described by Laemmli (11).

Plasma samples were also passed through a series of Amicon membranes, the initial ultrafiltration step employing a 1 kDa (YM-2) membrane. The retentate was reconstituted in distilled water and heated for 10 min at 70°C in the presence of 4% beta-mercaptomethanol and 1 mol/L formic acid. The solution was cooled down and subsequently placed on a 30 kDa (YM-30) membrane. The resulting filtrate, containing the dissociated protein, was lyophilized and subjected to further purification.

The dissociated protein was adsorbed onto a SEP-PAK C-18 cartridge. Interfering compounds, e.g., small peptides, hydrophobic substances, etc., were retained on the SEP-PAK C-18 cartridge. The protein of interest was eluted off the SEP-PAK C-18 cartridge with distilled water. This fraction was lyophilized, reconstituted in 1 mL of distilled water, and subsequently separated on Sephadex G-75. The plasma preparation was eluted off the column with 10 mmol/L ammonium acetate, pH 6.5. Fractions containing the Na-K-ATPase inhibitory material (12 kDa protein), which eluted after the albumin peak, were pooled, lyophilized, and subjected to a series of assays.

**Figure 2:**
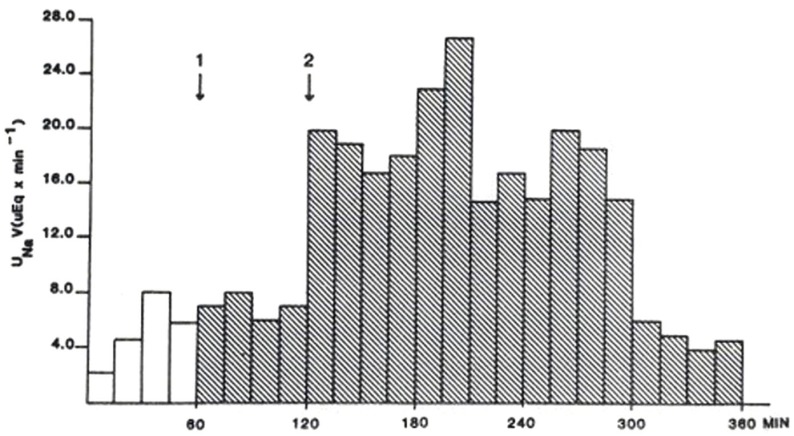
**Rat natriuretic bioassay of semi-purified 12 kDa protein representing 2.5 mL of original plasma**. First arrow designates time of injection of control solution. The second arrow designates the time of injection of the 12 kDa protein. From Ref. (10) with permission from the editors.

Duplicate bioassay procedures for the natriuretic response of the 12 kDa protein were performed according to the method described by Purdy et al. (12). Outcomes were averaged. Results are displayed in Figure 2.

An Econosphere C-18 column was packed with Econosphere C-18 silica, 5 μm particle size. The reversed phase C-18 column was equilibrated with triple distilled water. The LMW plasma Na-K-ATPase inhibitor (p-NKAI) was eluted off the column with a linear acetonitrile gradient (0–100% over a period of 30 min). The eluate was continuously monitored at 210 nm. One minute fractions were collected, lyophilized, and subsequently tested for the presence of Na-K-ATPase inhibitory activity.

**Figure 3:**
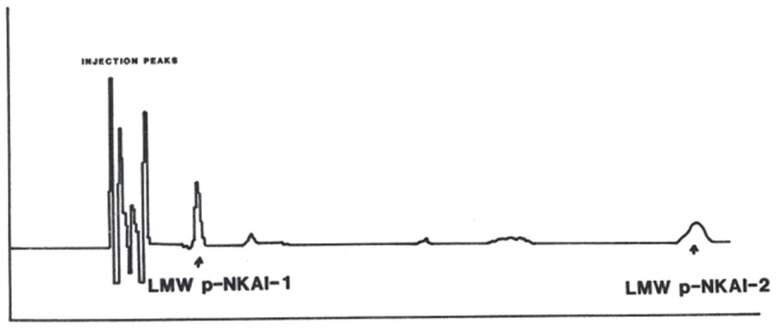
**Elution of semi-purified LMW p-NKAI, monitored electrochemically**. Arrows indicate the loci of p-NKAI-1 and p-NKAI-2. From Ref. (10) with permission from the editors.

P-NKAI was further purified by HPLC separation combined with electrochemical detection using a Model 5100A Coulocomb Detection System. On reversed phase C18 chromatography, p-NKAI appeared at 4% acetonitrile, co-eluting with a urinary inhibitor. P-NKAI was ultrafiltrable through an Amicon YM-05 membrane and thus has a presumed molecular weight of less than 500 Da. Rechromatography of active fractions on a 3 μm C-18 column monitored electrochemically yielded two active compounds, p-NKAI-1 and p-NKAI-2, both of which were inhibitors of the Na-K-ATPase enzyme system (Figure 3). P-NKAI-1 caused 50% inhibition and p-NKAI-2 caused 8% inhibition of Na-K-ATPase in a volume of inhibitor corresponding to 187 μL of original plasma. The remaining fractions were without inhibitory activity.

**Figure 4:**
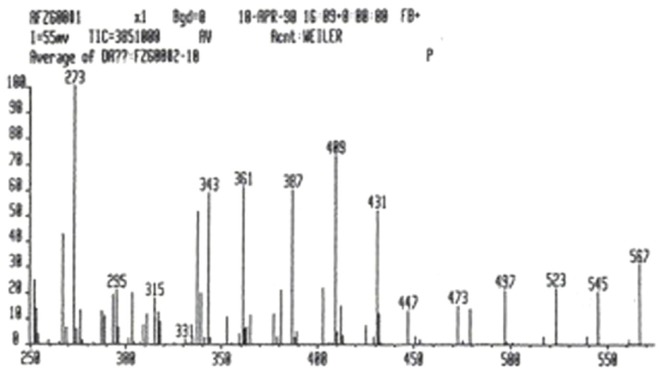
**Mass spectroscopy analysis of p-NKAI-1**. Note peaks at masses 409, indicating hydrogen adduct, 431, indicating sodium adduct, and 447, indicating potassium adduct. The mass of p-NKAI-1 is, therefore, 408. From Ref. (10) with permission from the editors.

The mass spectrum of p-NKAI-1 showed a fairly intense protonated molecular ion at mass 409 and also the sodium and potassium adduct ions at masses 431 and 447, respectively. This would indicate that the molecular weight of p-NKAI-1 is 408 Da (Figure 4).

A purified hog cerebral cortex Na-K-ATPase preparation was employed for Na-K-ATPase and K-pNPPase inhibition assays. The tubes were preincubated with either the 12 kDa protein or the purified LMW plasma factor for 5 min at 37°C. The enzymatic reaction was initiated by adding 0.025 mL enzyme preparation (25 mg/mL). The reaction was stopped by adding 1.0 mL ice cold 10% trichloroacetic acid after an incubation time of 15 min. After centrifugation, 0.5 mL of supernatant was assayed for inorganic phosphate according to the procedure described by Fisk and Subbarow (13). Both the 12 kDa protein and the LMW plasma factor (p-NKAI) were shown to inhibit the Na-K-ATPase and K-pNPPase enzyme systems in a dose-related manner, analogous to ouabain. The IC_50_ for inhibition of Na-K-ATPase by p-NKAI corresponds to 8 × 10^−7^ mol/L ouabain equivalents.

P-NKAI-1 was also tested for its vasoactive properties according to the procedure described by Purdy and Weber (14). Isolated femoral artery segments from New Zealand White rabbits were sectioned into 3 mm segments, then mounted in a 30 mL tissue bath containing Krebs-bicarbonate solution aerated continuously with 95% O^2^/5% CO_2_ at 37°C. Subsequently, p-NKAI-1 was assayed for its vasoactive behavior in the presence and absence of norepinephrine. A dose–response curve was established for p-NKAI-1; the concentration of p-NKAI-I yielding 1% contractile response was selected for the studies of synergy with norepinephrine. One hundred microliters of p-NKAI-1 produced a 1% contractile response, 300 μL produced a 5% contractile response and 600 μL of p-NKAI-1 produced an 18% contractile response. Similarly, the addition of 100 μL of p-NKAI-1 to a bath containing 10^−8^ mol/L norepinephrine increased the contractile response from 60 to 86%.

The dose–response curve for Na-K-ATPase inhibition of the semi-purified 12 kDa protein paralleled the dose–response curve for ouabain; 50% inhibition of Na-K-ATPase, corresponding to 5 × 10^−6 ^mol/L ouabain, was produced by the 12 kDa inhibitor in a fraction containing 2.7 mg/mL Lowry protein.

The ^3^H ouabain displacement assay revealed that the 12 kDa protein fraction displaces ^3^H-ouabain from its receptor in a dose-related manner, similar to ouabain. There was no cross-reactivity with digoxin antibody.

## Comparison of 12 kDa Protein, Marinobufagenin, and Ouabain in Various Disease States

**Figure 5:**
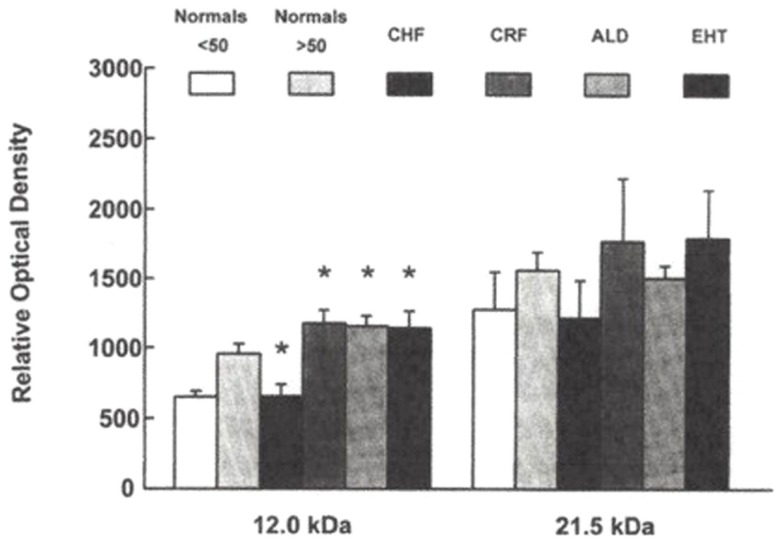
**Relative optical densities of 12 and 21 kDa bands**. *p < 0.05 for CRF, Aldo, EHT compared to normals < 50 and for CHF compared to normals > 50. From Ref. (14) with permission.

**Figure 6:**
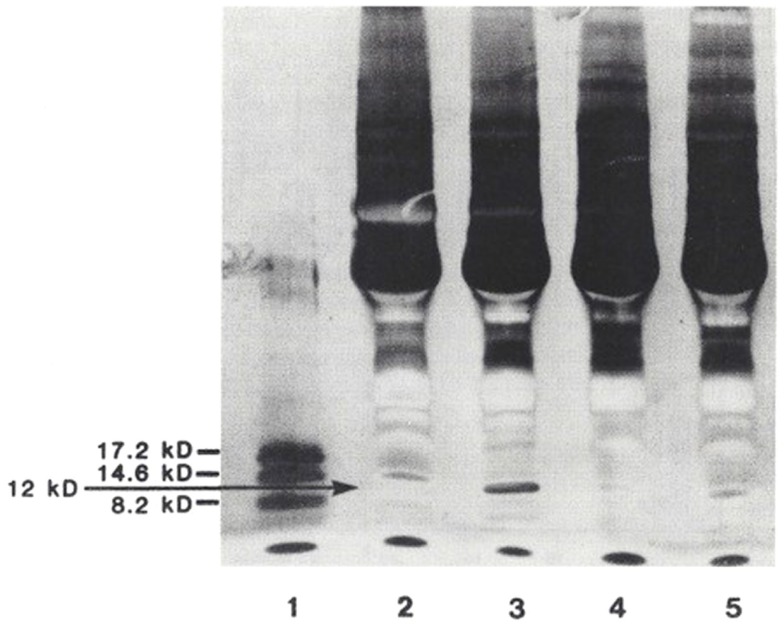
**SDS-PAGE separation of protein bands from the plasma of patients with various volume-related disease states**. Myoglobin protein markers of 17.2, 14.6, and 8.2 kDa are shown in lane 1. Lane 2: normal control. Lane 3: hyperaldosteronism. Lane 4: congestive heart failure, pre-treatment. Lane 5: congestive heart failure, post-treatment. These gel separations are from the plasma of individual patients. From Ref. (10) with permission from the editors.

In a third study (15), plasma from 101 patients were examined [25 normals (N) < age 50, 13N > age 50, 7 with acute CHF, 24 with chronic renal failure (CRF), on dialysis, 5 with idiopathic hyperaldosteronism (PA), and 27 with essential hypertension, untreated (EHT)]. Plasma was extracted with 32% acetonitrile, and analyzed by fluoroimmunoassay (DELFIA) for marinobufagenin and ouabain. In addition, from 32 patients (6N < 50, 6N > 50, 5 CHF, 5 CRF, 6 EHT, and 4 PA), SDS gradient gels were obtained. The 12 kDa bands were extracted, analyzed for Na-K-ATPase inhibition, marinobufagenin, and ouabain, and compared to 14 and 21 kDa bands. Marinobufagenin was found to be elevated in CRF, EHT, PA, and CHF. Ouabain was increased only in PA. When the relative optical densities of 12 and 21 kDa bands were contrasted, CRF, PA, and HT were found to be increased and CHF to be decreased in the 12 kDa band, with no discernible changes in the 21 kDa bands (Figure 5). Following extraction of the bands, Na-K-ATPase inhibitory activity measured 38% in 16 pooled 12 kDa bands, with essentially no activity found in the 14 kDa or 21 kDa bands. SDS-PAGE separation of plasma proteins confirmed that the 12 kDa band was elevated in primary aldosteronism, diminished in CHF, with return toward normal after treatment (Figure 6). Thus, only the 12 kDa band possessed all of the attributes of natriuretic hormone.

## Discussion

Following an initial flurry of activity, which utilized natriuresis as an index of hormone activity, most subsequent studies of natriuretic hormone have utilized Na-K-ATPase inhibition as a more rapidly obtained index (1–3). Digitalis-like (EDLF) or ouabain-like activities (OLF), measured by radioimmunoassay, were also initially employed as measures of natriuretic hormone. But digoxin-like immunoreactivity was found to be non-specific (16), while the radioimmunoassay for OLF did not prove reliable when measured by HPLC followed by ELISA (17), or by ultrasensitive UPLC-MS/MS (18). The lower limit of quantification by the latter method was 1.7 pmol/L, while ouabain was non-detectable. The suggestion that the presence of endogenous ouabain in human beings is non-detectable has been vigorously debated by Blaustein (19). For the moment, therefore, we must consider this an unresolved matter. Thus, we are left with the Na-K-ATPase inhibition assay as presumably the most reliable as well as the most rapid assay of EDLF activity.

The remaining ouabain-like hormone, which has been suspected to be the putative natriuretic hormone, is marinobufagenin (20), for which studies of activity in several diseases have appeared, including volume-expanded normals (20), CHF (21), CRF (22), essential hypertension (23), primary aldosteronism (15), and pre-eclampsia (24). The one noteworthy discrepancy between natriuretic hormone determined by marinobufagenin radioimmunoassay and natriuretic hormone, as determined by the Na-K-ATPase assay, are the findings in CHF [see above and Ref. (25)]. Urinary sodium values are low in CHF, LMW urinary Na-K-ATPase inhibitors are also lower than normal (25), and arterial central volume is diminished rather than increased. Kramer and Kruck (26) found that a natriuretic substance present in an ultrafiltrate of normal urine from volume-expanded individuals was absent in the urine of patients with edema related to cirrhosis with ascites or with nephrotic syndrome, edematous states physiologically similar to CHF. Furthermore, they also demonstrated that plasma and urine fractions of normal individuals following Sephadex G-25 separation consistently reduced short-circuit current when applied to the serosal surface of frog skin (anti-natriferic effect) (26), whereas plasma and urine fractions from patients with edema lacked this effect. In addition, we have shown previously that although the LMW Na-K-ATPase inhibitor in human urine has less activity than normal in CHF, the activity reverts toward normal as CHF improves (25).

The radioimmunoassay for marinobufagenin has been recently validated by high resolution mass spectrometry but has been measured only in CRF, where it is elevated (27). Until the CHF results are similarly verified, it is not possible to be sure that radioimmunoassay results for marinobufagenin in disease states other than CRF also reflect the true status.

We suspect that the HMW Na-K-ATPase inhibitor may be a carrier protein for the LMW inhibitor since the latter can be split off by use of beta-mercaptoethanol, an agent known to cleave S-S bonds, plus heat and formic acid, properties employed by Lindner et al. (28) to dissociate oxytocin and vasopressin from their neurophysin carrier. It is also pertinent that Morich and Garthoff (5) found that both salt-sensitive (DS) and salt-resistant (DR) rats displayed two protein bands in their plasma on SDS-PAGE, in the molecular weight range of 14–15 kDa. When DS rats were given salt and developed hypertension, the upper band diminished but the lower band became more intense. The difference in molecular weight between the two bands was estimated to be between 300 and 400 Da. Mass spectrometry of the first of the LMW inhibitors in the present study (NKAI-1) revealed a molecular weight of 408 Da, as shown by the hydrogen adduct of 409 Da, the sodium adduct of 431 Da, and the potassium adduct of 447 Da. The molecular weight of 408 is identical to that described by Kerek (29), a biochemist, for an initially identified macrocylic derivative of inorganic carbon suboxide, which is a natriuretic, Na-K-ATPase inhibiting compound derived from plant tissue. We look forward with interest to the comparison between Kerek’s 408 Da compound and the 408 Da compound discussed in this review.

The HMW compound of the present dissertation was previously referred to as “hypertension-associated protein” by Van de Voorde et al. (7). These authors claimed an approximate molecular weight of 15 kDa for the compound they isolated by chromatography after reduction of the disulfide bridges of the precursor 105 kDa protein molecule with beta-mercaptoethanol. In a prior study of plasma proteins in essential hypertension, utilizing SDS-PAGE to separate the plasma proteins, Nardi et al. (4) had earlier reported a 14 kDa protein present in such patients but not in patients with hypertension secondary to renovascular hypertension or renal parenchemal disease. Cloix et al. (6) had reported a 13 kDa protein in the plasma of hypertensive human beings and rats. Thus, we are left with four studies that purport to show either 12, 13, 14, or 15 kDa proteins in the plasma of human beings with essential hypertension but not in normal controls or possibly in renovascular hypertension or hypertension with CRF. What could this protein be? In the present study, we have referred to the 12 kDa protein as a “carrier protein” because the Na-K-ATPase inhibitor can be split off by heat and formic acid. But are there alternatives?

To explore this question in all of its ramifications, it is first necessary to review what has been learned about the “other” natriuretic system, namely the natriuretic peptides. Following the initial description of natriuretic peptides by deBold and associates in 1961 (30), it has been found that there are at least three natriuretic peptides released from the hypothalamus and cardiac tissue – atrial natriuretic factor (ANF), B-type natriuretic factor (BNF), and C-type natriuretic factor (CNF). All occur initially as pre-prohormones, which are degraded to prohormones and then finally to the active peptides (31).The molecular weight of the proANF, a circulating compound (32), has been described as 14 kDa (33). Is it possible that pro-ANF is identical to the hypertension-associated protein described by Van de Voorde et al. (7), Nardi et al. (4), Cloix et al. (6), and the present study? A suggestion that this may be the case comes from Melander et al. (34) who described in offspring of hypertensive human beings a strong correlation between salt sensitivity, as defined by the difference in sodium excretion while on a low salt diet and then on a high salt diet, and plasma proANP levels.

Initially, it was thought that EDLF, endogenous digitalis-like factor, or OLF, ouabain-like factor, as the Na-K-ATPase inhibitor became known, could be distinguished physiologically from ANF by its Na-K-ATPase inhibiting property as well as its tendency to increase, rather than decrease, vasoconstriction when applied to isolated blood vessels (10). However, it was recognized by Górny et al. (35) that ANF does inhibit Na-K-ATPase in the rat renal medulla, but not in the rat renal cortex, where the proximal tubule is located. In contrast, Chiou and Vesely (36) reported that kaliuretic peptide, a fraction split off from ANF prohormone, inhibits both renal cortical and medullary Na-K-ATPase. However, these experiments employed rat renal tissue rather than hog cerebral cortex for assay of Na-K-ATPase and the inhibition in the two studies quoted resulted from indirect inhibition of Na-K-ATPase through effects of second messengers, namely, dopamine in the first study (35) and prostaglandin E_2_ in the second study (36). Thus, we may no longer be able to depend exclusively on the Na-K-ATPase assay to distinguish between ANF and EDLF. On the other hand, we can still depend on both the molecular weight and the direct vasoconstrictive (10) or vasodilatory (37) actions on isolated vascular smooth muscle preparations to distinguish between EDLF and ANF. The molecular weights for EDLF have been reported as varying between 360 and 620 Da (Table 1), while the molecular weights for ANF have been described as 3800 Da for rat ANF (38) and varying from 3000 Da (33) to 5499 Da (39) for human ANF.

**Table 1:** **Comparison of sources and molecular weights of various EDLFs**.

Author	Source	Molecular weight (daltons)	Reference
Bricker et al.	Human uremic urine	360	(40)
McKinnon et al.	Human placenta	370	(41)
Kramer et al.	Na-loaded normal human urine	391	(38)
Cloix et al.	Normal human urine	431	(42)
Weiler et al.	Hypertensive human plasma	408	(10)
Kerek	Plant tissue	408	(29)
Tamura et al.	Pig urine	620	(43)

Haupert (44) in 1988 first posed the question as to whether there is an interrelationship between natriuretic peptides and EDLF or OLF. That the interrelationship exists can no longer be in doubt. It has long been recognized that both ANF and EDLF are released from the hypothalamus (45, 46), and in fact from the AV3V region (47). Lesions produced in the AV3V region prevent the natriuresis following isotonic saline volume expansion in experimental animals. Furthermore blood drawn following expansion failed to show an anti-natriferic effect in the toad bladder in contrast to control animals, implying interference with release of the Na-K-ATPase inhibitor. The perfusate from incubation of fragments of rat brain inhibited the Na, K pump by a 77% reduction of ouabain-sensitive ^86^Rb uptake into human erythrocytes. This did not occur when ANF was given intravenously before sacrifice of the test animals (48). ANF injected into lateral cerebral ventricles releases an Na-K-ATPase inhibitor measured as above in cultured aortic smooth muscle cells (49). Ouabain and digoxin, cardiotonic steroids resembling EDLF and OLF, increase ANF secretion by rat atrial cardiocyte superfusions (50). Liu et al. (51) also employed the perfused beating rabbit atria model to show that ouabain significantly increased ANF secretion in a dose-dependent manner, indicating that the interrelationship between Na-K-ATPase inhibitors and ANF can proceed in both directions.

Finally, in an elegant experiment performed by Morgan et al. (52), using extracts from cultured rat hypothalamic cells separated on Sephadex G-25, a sodium transport inhibitor could be recovered from the post-salt fraction as indicated by three assays: (1) inhibition of transport in human erythrocytes, (2) displacement of ^3^H ouabain from its binding site, and (3) direct inhibition of canine Na-K-ATPase. Could pro-ANF and EDLF be co-secreted by the hypothalamus in response to volume expansion or as an indicator of pre-disposition to essential hypertension? A suitable way to settle this question would be to perform immunoassays for pro-ANF and ANF on the 12 kDa protein of the present experiment. For this reason, I would again implore currently active investigators to separate the 12 kDa protein from human hypertensive plasma and test it for pro-ANF and ANF immunoreactivity.

## Conflict of Interest Statement

The author declares that the research was conducted in the absence of any commercial or financial relationships that could be construed as a potential conflict of interest.
